# Endonuclease G promotes mitochondrial genome cleavage and replication

**DOI:** 10.18632/oncotarget.24822

**Published:** 2018-04-06

**Authors:** Rahel Stefanie Wiehe, Boris Gole, Laurent Chatre, Paul Walther, Enrico Calzia, Miria Ricchetti, Lisa Wiesmüller

**Affiliations:** ^1^ Department of Obstetrics and Gynecology, Ulm University, Ulm, 89075, Germany; ^2^ Present address: Centre for Human Molecular Genetics and Pharmacogenomics, Medical Faculty, University of Maribor, Maribor, SI-2000, Slovenia; ^3^ Department of Developmental and Stem Cell Biology, Institute Pasteur, Stem Cells and Development, 75724 Cedex 15, Paris, France; ^4^ Team Stability of Nuclear and Mitochondrial DNA, Unit of Stem Cells and Development, CNRS UMR 3738, 75724 Cedex 15, Paris, France; ^5^ Central Facility for Electron Microscopy, Ulm University, Ulm, 89081, Germany; ^6^ Institute of Anesthesiological Pathophysiology and Process Engineering, Ulm University Hospital, Ulm, 89081, Germany

**Keywords:** endonuclease G, oxidative damage, base excision repair, mitochondrial DNA degradation

## Abstract

Endonuclease G (EndoG) is a nuclear-encoded endonuclease, mostly localised in mitochondria. In the nucleus EndoG participates in site-specific cleavage during replication stress and genome-wide DNA degradation during apoptosis. However, the impact of EndoG on mitochondrial DNA (mtDNA) metabolism is poorly understood. Here, we investigated whether EndoG is involved in the regulation of mtDNA replication and removal of aberrant copies. We applied the single-cell mitochondrial Transcription and Replication Imaging Protocol (mTRIP) and PCR-based strategies on human cells after knockdown/knockout and re-expression of EndoG. Our analysis revealed that EndoG stimulates both mtDNA replication initiation and mtDNA depletion, the two events being interlinked and dependent on EndoG's nuclease activity. Stimulation of mtDNA replication by EndoG was independent of 7S DNA processing at the replication origin. Importantly, both mtDNA-directed activities of EndoG were promoted by oxidative stress. Inhibition of base excision repair (BER) that repairs oxidative stress-induced DNA damage unveiled a pronounced effect of EndoG on mtDNA removal, reminiscent of recently discovered links between EndoG and BER in the nucleus. Altogether with the downstream effects on mitochondrial transcription, protein expression, redox status and morphology, this study demonstrates that removal of damaged mtDNA by EndoG and compensatory replication play a critical role in mitochondria homeostasis.

## INTRODUCTION

**Figure 1:**
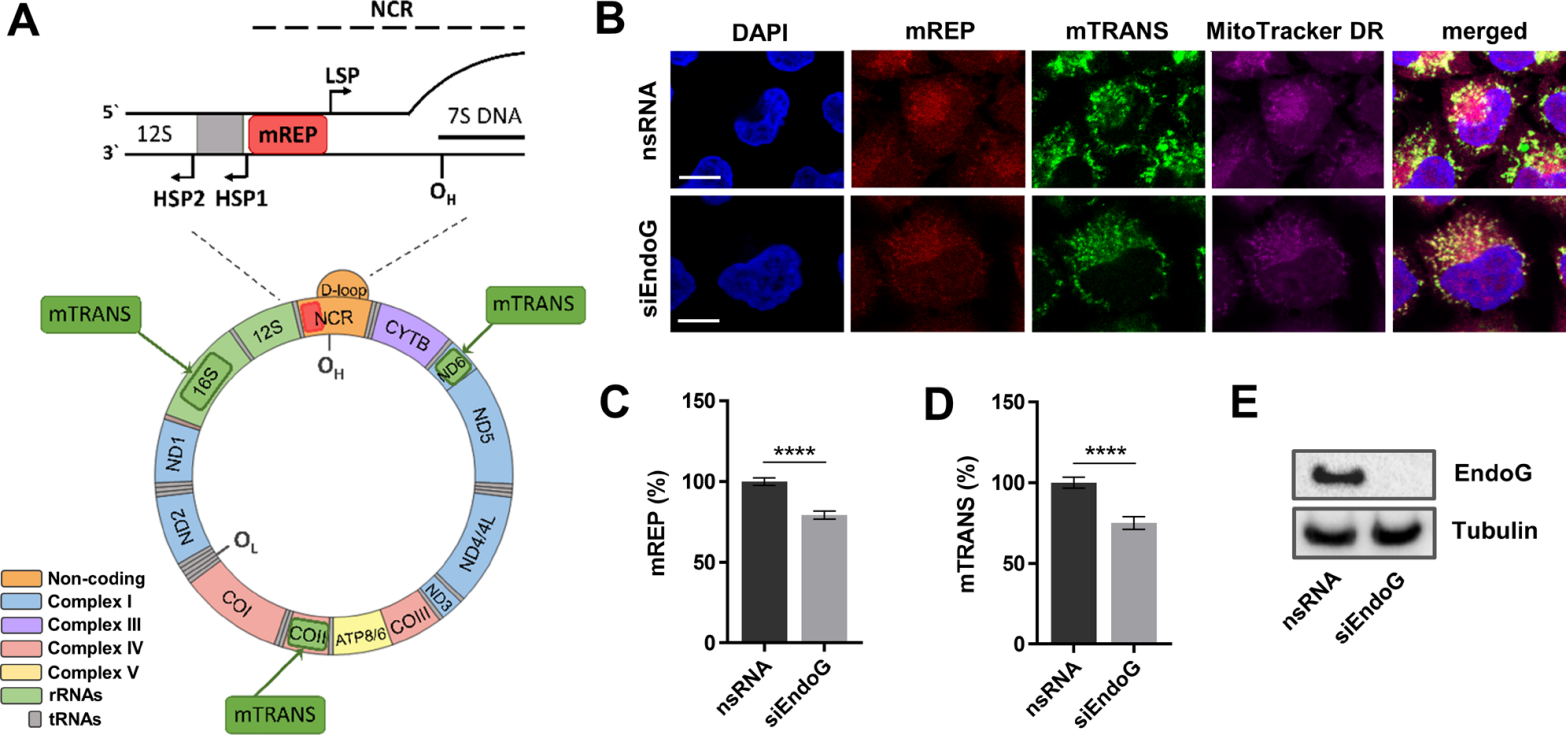
Assessment of EndoG's function in initiation of mitochondrial O_H_ replication and mitochondrial transcription (**A**) Organization of the human mitochondrial genome. Recognition sites of mREP and mTRANS probes for FISH labelling with mTRIP are indicated with red and green boxes, respectively. HSP and LSP - heavy - and light-strand promoter, O_H_ - origin of heavy strand replication, NCR - non-coding region (adopted from [7]). (**B**) Representative confocal microscopic images of HeLa cells stained with DAPI and MitoTracker DR (250-1000 nM, 1 h) and labelled with mREP and mTRANS probes 48 h after EndoG knockdown with siRNA. Scale bars = 10 μm. Fluorescence-based quantification of (**C**) mREP (initiation of mtDNA replication near O_H_) and (**D**) mTRANS (mitochondrial transcription); *n* = 196–218 cells from 2 independent experiments. Data are expressed as mean ± SEM (^****^P < 0.0001; non-parametric Mann-Whitney test for unpaired samples). (**E**) Western blot analysis to validate efficient knockdown of EndoG.

Mitochondria harbour their own small circular DNA (16569 bp in size in humans), which encodes 13 proteins, essential for the functionality of the electron transport chain (ETC), 22 tRNAs as well as 2 ribosomal RNAs (Figure 1A). mtDNA molecules contain a non-coding region (NCR), where the light and heavy strand promoters (LSP and HSP) as well as the origin of heavy strand replication (O_H_) are located [1, 2]. To date two prevalent models describe replication in mammalian mitochondria: First, the strand-displacement replication model, which was suggested in the 1970s and postulates that heavy-strand synthesis starts within the displacement-loop (D-loop) near or at O_H_ and proceeds unidirectionally until after two thirds the light-strand DNA synthesis is initiated at O_L_ (origin of light strand replication) on the other strand [3]. According to this model mtDNA replication results in single-stranded intermediates, which are coated and stabilised by the mitochondrial single-stranded DNA binding protein (mtSSB). Second, the RITOLS (Ribonucleotide Incorporated ThroughOut the Lagging Strand) model also proceeds via such heavy-strand intermediates, however, the light-strand becomes temporarily hybridised to RNA, which is subsequently removed prior DNA synthesis [4]. According to this last model, also-called the bootlace mechanism, this RNA is derived from processed transcripts, threaded onto the displaced light-strand template to aid protecting single-stranded intermediates from damage and nucleolysis [5]. The occurrence of each of these mechanisms is still a matter of debate. Possibly different replication modes are utilised for specific requirements, such as under physiological conditions versus recovery after mtDNA depletion [6]. Both models include in a fraction of initiation events the production of a short fragment of DNA that terminates about 700 nt downstream of the replication origin O_H_, the 7S DNA, that remains associated with the replication structure forming a triple-stranded DNA. The role of 7S DNA is still unclear [7].

According to these specific requirements the mtDNA replication machinery is distinct from those in the nucleus. Mainly three key proteins, encoded by nuclear genes, form the mitochondrial replisome. The mtDNA polymerase γ (POLγ) [8], the replication helicase TWINKLE, which unwinds double stranded mtDNA in 5′ -> 3′ direction [9], and mtSSB, that may protect mtDNA during the replication process and additionally stimulates the activities of POLγ [10] and TWINKLE [9]. In humans mtDNA replication primers are formed via transcription from the LSP. The transition from transcription to replication has been mapped to the conserved sequence block (CSB) II region, which is located approximately 100 nucleotides upstream of O_H_. It is still unclear which factors are involved in the termination of transcription in CSBII [1]. It has been claimed that primers are removed by ribonuclease H1 (RNASEH1) [11] and subsequently nascent DNA is processed from CSBII down to O_H_ by the mitochondrial genome maintenance exonuclease-1 (MGME1), possibly to ensure a proper ligation site by DNA ligase III after completion of replication of both ends of newly synthesised DNA [1].

In 1993, Côté and Ruiz-Carrillo [12] suggested that the major mitochondrial magnesium/manganese-dependent nuclease, EndoG, is involved in the initiation of mtDNA replication by RNA primer maturation. In this report it was proposed to preferentially target the C/G-rich CSBII sequence, thereby acting as RNase H. However, this hypothesis was not pursued further. Moreover, two independent EndoG-depleted (EndoG^−/−^) mouse models did not show an effect on mtDNA copy number, structure or mutation rate [13, 14]. More recently McDermott Roe *et al.* [15] reported that EndoG regulates mitochondrial mass, function and reactive oxygen species (ROS) production in cardiac tissue of one of these EndoG^−/−^ mice and modulates the expression of mitochondrial proteins. In the light of these observations, it is necessary to revisit EndoG's role in mitochondrial biogenesis. EndoG is encoded by nuclear genes [16] and targeted to mitochondria as an inactive precursor protein by a mitochondrial targeting sequence [17]. Subsequently, this sequence is cleaved off, giving rise to the mature form of the nuclease (~29 kDa). EndoG is well-known to participate in nuclear genome degradation during programmed cell death [18, 19], but was recently discovered to also cleave the breakpoint cluster region in the Mixed-lineage leukemia (*MLL*) gene during replication stress [20]. EndoG is directly regulated by Estrogen-related receptor alpha (ERRα) and Peroxisome proliferator-activated receptor gamma coactivator 1α (PGC1α), which are known to be key regulators of mitochondria [15]. For a long time EndoG was thought to localise exclusively within the mitochondrial intermembrane space [21], but later on it was also found to be bound to the mitochondrial inner membrane [22]. Its proximity to the mitochondrial matrix, where mtDNA is located, and the ability to cleave RNA, DNA and hybrid molecules makes EndoG an excellent candidate to be involved in mtDNA replication and mtDNA metabolism [12, 16, 23–25]. This notion was supported by experimental evidence demonstrating direct interaction of EndoG with mtDNA, in a mitochondrial transcription factor A (TFAM)-like fashion [15]. Interestingly, the expression of EndoG in different rat tissues seems to correlate with the levels of oxidative phosphorylation (OXPHOS) [26]. Moreover, nicks in one DNA strand or other types of DNA damage enhance the cleavage susceptibility of EndoG, suggesting a role in the removal of oxidatively damaged mtDNA molecules [26, 27].

Several evidences thus point to EndoG`s involvement in physiological processes acting on mtDNA, but the precise function of this protein is still unknown. Since qualitative or quantitative abnormalities of mtDNA are associated with several human disorders such as neuromuscular and neurodegenerative mitochondriopathies, diabetes, cardiovascular diseases, cancer and aging [28–33], a better understanding of factors involved in mtDNA maintenance is indispensable. Therefore, the main aim of this study is to investigate the specific role of EndoG in mtDNA replication and cleavage impacting on mitochondrial genome integrity.

## RESULTS

### EndoG promotes mtDNA replication initiation independently of 7S DNA production

In order to analyse a potential role of EndoG in mtDNA replication and transcription at the single-cell level, we used the established mTRIP imaging protocol [34–36]. The principle of mTRIP is a combination of RNA and DNA fluorescence *in situ* hybridization (FISH), relying on the following probe design: the mREP probe is localised in the upstream neighbourhood of the main replication origin O_H_ (between the two divergent promoters LSP and HSP) and recognises non-transcribed DNA that becomes accessible during initiation of mtDNA replication; this signal includes the production of extended mtDNA replication as well as abortive 7S structures. The mTRANS probe is a mix of three probes indicating mitochondrial transcription by the labelling of processed mitochondrial RNAs and unprocessed immature mitochondrial RNAs encoded at different positions within the mitochondrial genome. A schematic illustration of the human mitochondrial genome and the probes used in mTRIP are represented in Figure 1A. When applying mTRIP on HeLa cells after silencing EndoG with siRNA, we observed a significant reduction in the fluorescence signal indicating diminished initiation of mtDNA O_H_ replication (21% mREP, *p <* 0.0001) and transcript levels (25% mTRANS, *p <* 0.0001) in comparison to control cells with nsRNA (Figure 1B–1D), without a decrease in mitochondrial content indicated by MitoTracker DR and citric acid synthase activity (Supplementary Figure 1A, 1B). Western blotting validated that siRNA efficiently reduced the protein level of EndoG (Figure 1E). Whole cell lysates were analysed, since during unperturbed growth the majority of EndoG is located in the mitochondria (Supplementary Figure 1C). Notably, when we generated HeLa clones stably expressing EndoG-specific small hairpin RNA (shRNA), we observed phenotypic differences between individual knockdown clones already during the earliest cell passages (data not shown). To avoid clonal heterogeneity, we therefore focused on functional analysis after transient knockdown.

**Figure 2:**
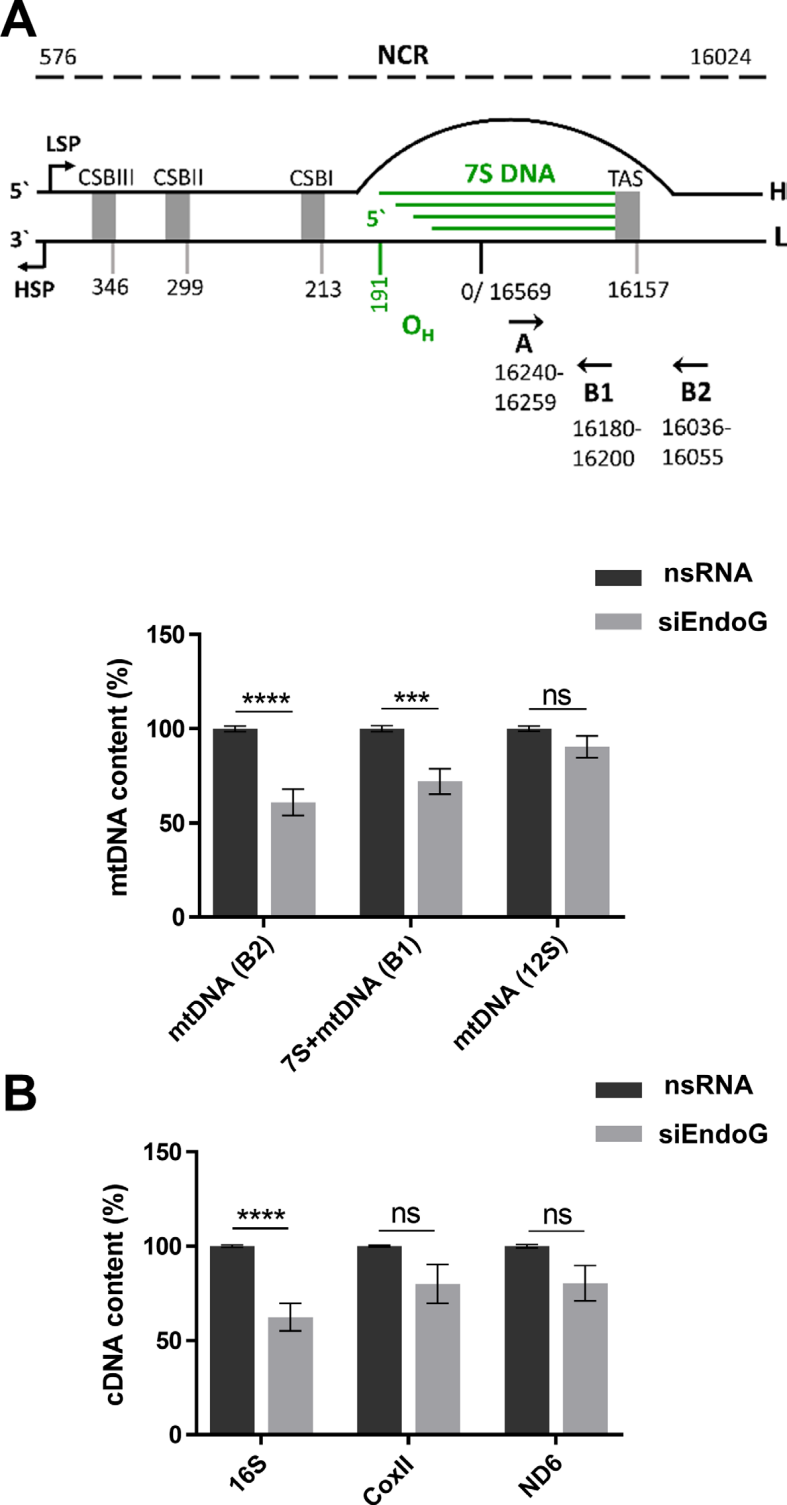
Examination of a potential role of EndoG in 7S DNA production and regulation of transcription HeLa cells were cultured in glucose medium and transfected with control nsRNA or siRNA targeting EndoG (siEndoG) and cultivated for 48 h. (**A**) Upper panel: schematic representation of the NCR including the D-loop. H - heavy strand, L - light strand, HSP and LSP - heavy - and light-strand promoter. CSB - conserved sequence block, TAS - termination associated sequences, O_H_ - origin of heavy strand replication. Primer positions for qPCR indicated with black arrowheads (reference: NC_012920.1, Gene Bank). 7S DNA and mtDNA content was determined using the same forward primer A and two specific reverse B primers. Primer B1 is located within 7S DNA, whereas B2 binds more downstream beyond 7S DNA (adopted from [7]). Lower panel: qPCR on total genomic DNA; 12S mtDNA level indicated full-length mtDNA content and the nuclear encoded 18S rRNA DNA level served as internal control for data normalisation. *n* = 8–12 from 3-4 independent experiments; (**B**) RT‐qPCR analysis of mitochondrial genes, which are targets of mTRANS labelling; the nuclear encoded TBP gene served as internal control for data normalisation. *n* = 9 from 3 independent experiments; Data are expressed as mean ± SEM (ns, not significant; ^*^P < 0.05; ^****^P < 0.0001; non-parametric Mann-Whitney test for unpaired samples).

Previous work has revealed that after initiation at or near O_H_ replication of the heavy strand commonly terminates approximately 700 nucleotides further downstream, giving rise to 7S DNA [7, 37]. When 7S DNA hybridises to the circular parental molecule, a characteristic triple-stranded D-loop structure is generated. Since an involvement of a protein binding to the termination associated sequences (TAS) region has been discussed in the context of 7S DNA formation [7], we examined whether EndoG is involved in this process. To this end we measured 7S DNA and mtDNA content by quantitative polymerase chain reaction (qPCR) using forward primer A targeting the 7S template region and two reverse primers, one targeting newly synthesised 7S DNA (B1) and the other one detecting synthesis beyond 7S DNA (B2) [38]. Primer pair A/B1 amplifies both 7S DNA as well as longer mtDNA (7S + mtDNA), whereas A/B2 produces a PCR product exclusively from synthesis beyond 7S DNA (mtDNA, Figure 2A). DNA synthesis beyond 7S DNA (mtDNA) significantly decreased by 39% after silencing EndoG (*p <* 0.0001). Similarly, EndoG knockdown caused a decrease of both 7S DNA and beyond (7S + mtDNA) by 28% (*p* = 0.0002) (Figure 2A lower panel). Accordingly, it is unlikely that EndoG is specifically involved in the regulation of 7S DNA production. However, when we additionally quantified the mtDNA level via qPCR specific for 12S DNA, which is located in the terminal part of the heavy-strand replication, we did not observe significant differences after EndoG silencing (*p* = 0.1402; Figure 2A lower panel). Consistent with this observation also mitochondrial mass, determined by MitoTracker DR staining, did not show any significant changes after EndoG knockdown under these conditions, i.e. in glucose medium (see above, Supplementary Figure 1A). Interestingly however, EndoG silencing induced morphological abnormalities of mitochondria as detectable via transmission electron microscopy (Supplementary Figure 1D). In specific, we noticed mitochondrial fragmentation from elongated, contiguous assemblies into smaller, round and disconnected units, suggesting functional deficits and/or a modulation of the mitochondrial fusion/fission machinery. Alterations in the complexes of the respiration chain I, II, III and V after silencing EndoG could be excluded via immunoblotting of the corresponding nuclear-encoded OXPHOS proteins (Supplementary Figure 1E).

In agreement with mTRANS data (detection of processed mitochondrial RNAs and unprocessed immature mitochondrial RNAs) we measured a significant reduction of the 16S cDNA level (38%, *p <* 0.0001), whereas smaller decreases in CoxII (20%, *p* = 0.2581) and ND6 (20%, *p* = 0.0939) cDNA levels after EndoG knockdown did not reach statistical significance (Figure 2B).

Taken together, and in agreement with our mREP data, EndoG stimulates mtDNA replication initiation at O_H_, not limited to 7S DNA production, and also stimulates mitochondrial transcription, at least at the level of ribosomal RNA. However, these EndoG effects do not consistently entail an increase in the mitochondrial mass despite an impact on mitochondrial morphology.

### The nuclease activity of EndoG is necessary for the regulation of replication initiation

**Figure 3:**
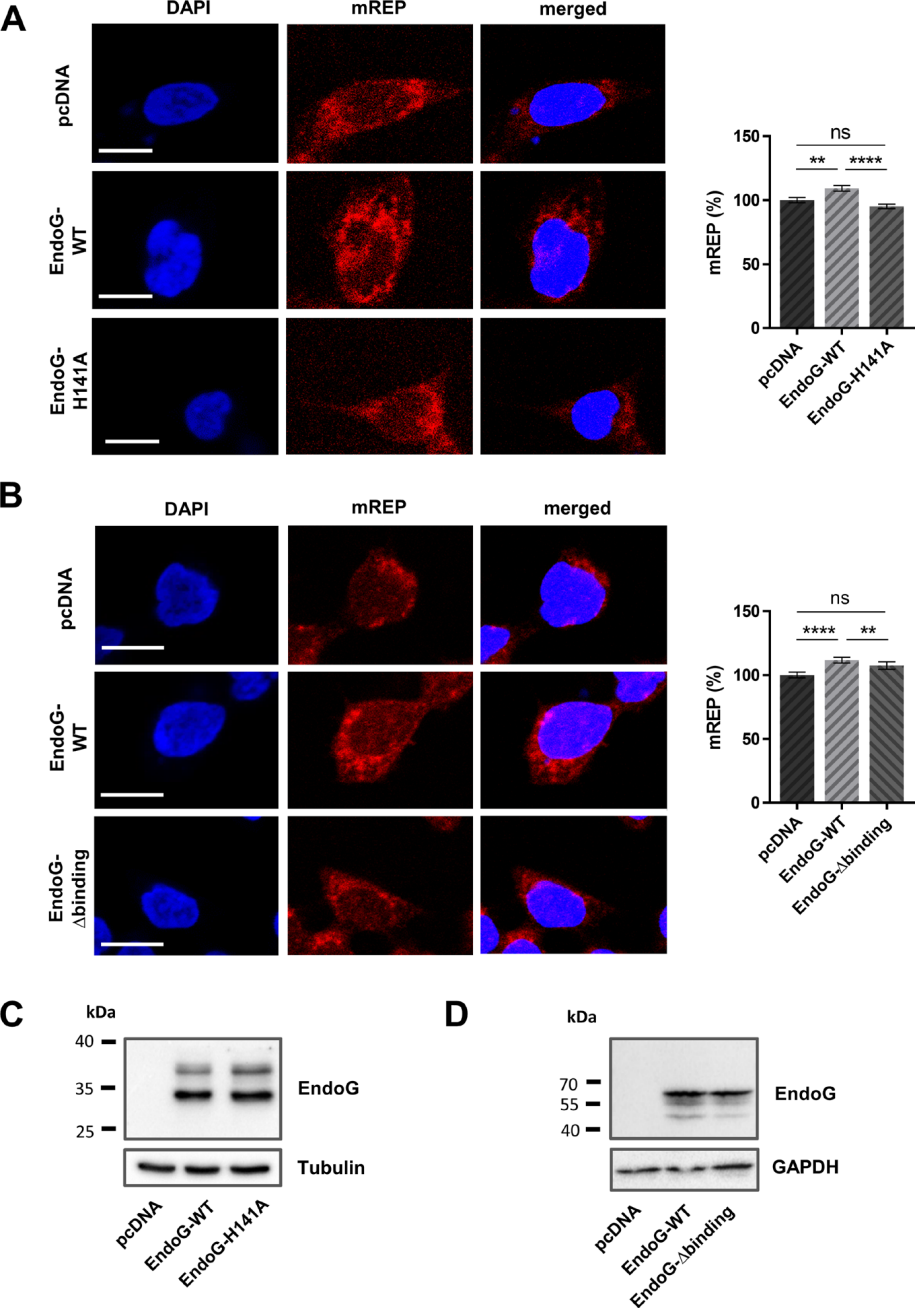
Impact of EndoG's nuclease and DNA binding activity for regulation of initiation of O_H_ replication Left panel: representative confocal microscopic images of HAP1 EndoG knockout cells transfected with empty control plasmid or a plasmid expressing EndoG-WT, (**A**) the catalytically inactive EndoG-H141A mutant or (**B**) the EndoG-Δbinding mutant, cultivated for 48 h, subsequently stained with DAPI and labelled with mREP probes. Right panel: Fluorescence-based quantification of mREP labelling. (A) *n* = 358–401 cells from 3 independent experiments for EndoG-WT versus EndoG-H141A expressing cells; (B) *n* = 299–336 from 2 independent experiments for EndoG-WT versus EndoG-Δbinding mutant expressing cells showing an increase after EndoG-WT expression; Data are expressed as mean ± SEM (ns, not significant; ^**^P < 0.01, ^****^P < 0.0001; non-parametric Mann-Whitney test for unpaired samples); Scale bars = 10 μm. (C, D) Western blot analysis to validate equivalent expression of the different EndoG variant proteins. Note that EndoG variants WT and H141A were expressed with Myc-DDK tag (**C**), whereas WT and Δbinding variants as HaloTag^®^ fusion proteins (**D**), with theoretical molecular masses of 29 kDa and 62 kDa, respectively, for the leader peptide-free mitochondrial proteins. Residual EndoG carrying the leader peptides with a theoretical molecular mass of 34 kDa were detectable for the Myc-DDK tagged proteins (C).

To analyse whether the nuclease activity of EndoG is relevant for mtDNA replication, we expressed either a wild-type (WT) or a catalytically inactive form of EndoG (EndoG-H141A) after CRISPR/Cas9-mediated EndoG knockout in HAP1 cells. EndoG-WT expression resulted in a significant 1.1-fold mREP signal increase in comparison to control cells, transfected with an empty vector (*p <* 0.0001, Figure 3A). Conversely, in cells expressing the nuclease-deficient EndoG mutant the mREP signal remained unchanged. Similarly, cells expressing a DNA binding deficient EndoG mutant (EndoG-R133A-H141A-R184A-R188A/EndoG-Δ-binding) displayed values for the initiation of replication signal (mREP) comparable to EndoG deficient controls (Figure 3B). Comparable expression of EndoG-WT, the catalytically inactive EndoG-H141A or EndoG-Δ-binding mutant was verified by Western blot analyses (Figure 3C, 3D). These results suggest that the DNA binding and nuclease activity of EndoG are essential for the regulation of replication initiation.

### EndoG is involved in mitochondrial genome cleavage

**Figure 4:**
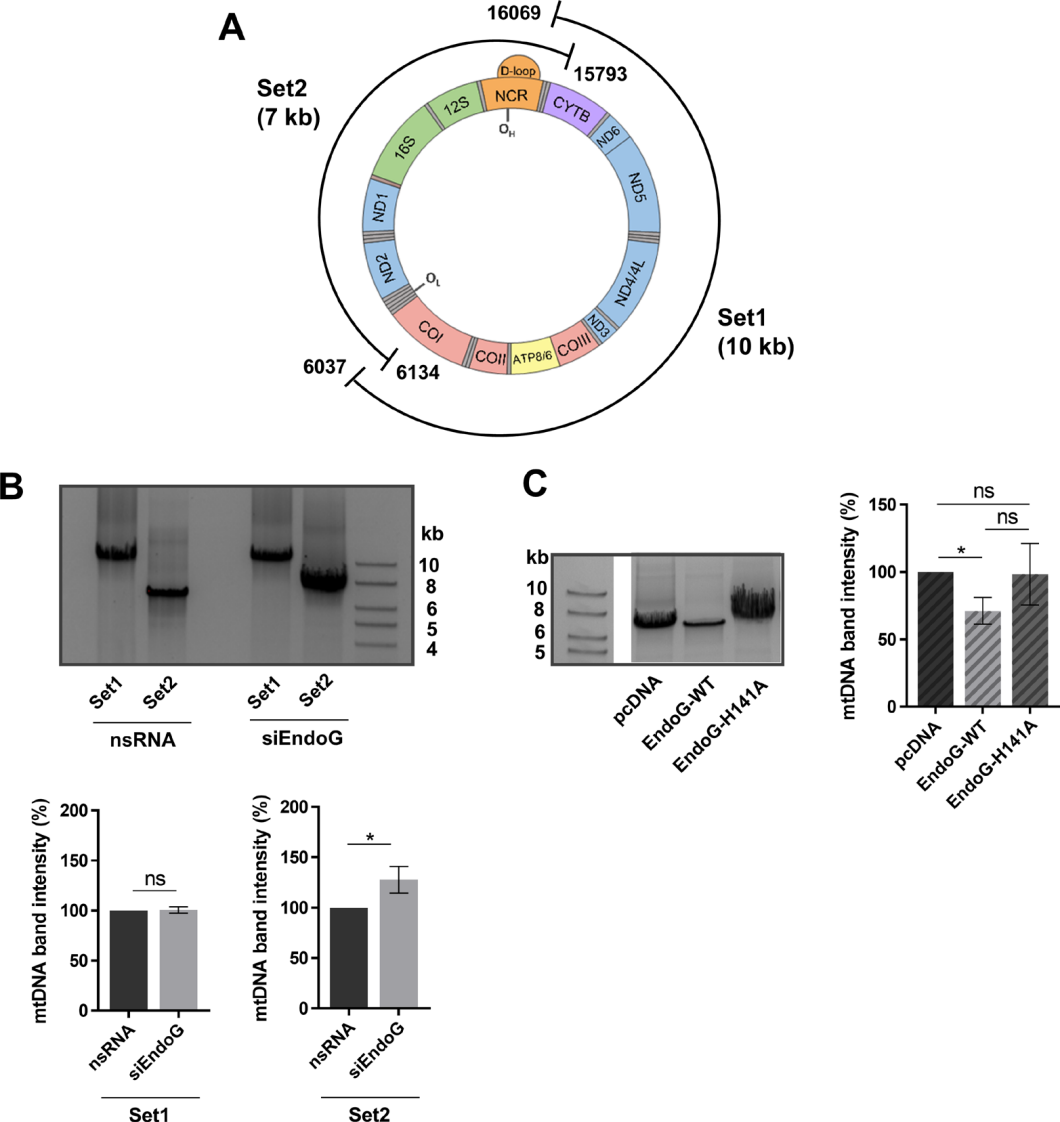
EndoG's role in mtDNA degradation (**A**) Scheme of positions of primer pairs for long-range PCR Set1 and Set2, which cover the whole mitochondrial genome in two overlapping segments. Set1 amplifies a fragment of 10,033 bp and Set2 a fragment of 6,910 bp (on a genome of 16,569 bp, NC_012920.1 GenBank). (**B**) Upper panel: representative image of agarose gel electrophoresis of long-range PCR on total genomic DNA isolated from HeLa cells grown in glucose medium and transfected with control nsRNA or siEndoG and subsequent cultivation for 48 h. Lower panel: quantification of 10 kb Set1 (*n* = 5 independent experiments with Hela cells grown in glucose medium) and 7 kb Set2 (*n* = 12 of 6 independent experiments with Hela cells grown in glucose or galactose medium) mtDNA band intensities after long-range PCR. (**C**) Left panel: representative image of agarose gel electrophoresis of long-range PCR on total genomic DNA isolated from HAP1 EndoG knockout cells transfected with a control plasmid or a plasmid expressing EndoG-WT or the catalytically inactive EndoG-H141A mutant for 48 h. The framed images were derived from the same agarose gel, obtained with the same exposure time. Right panel: quantification of Set2 mtDNA band; *n* = 8 independent experiments showing an decrease after EndoG-WT expression. Data are expressed as mean ± SEM (ns, not significant; ^*^P < 0.05; Wilcoxon matched-pairs signed rank test).

Mitochondrial DNA is heavily exposed to ROS due to its close proximity to the respiratory chain and is therefore highly susceptible to oxidative damage [39, 40]. Oxidative lesions in the nuclear and mitochondrial genome can be repaired by BER [41–43], but excessive or persistent damage is known to trigger mtDNA elimination through degradation [44, 45]. The enzymes involved in degradation of oxidatively damaged mtDNA in humans are still unknown. To examine a potential involvement of EndoG in mitochondrial genome degradation, we performed a previously established long-range PCR amplifying the entire mitochondrial genome with two sets of primers [46]. The positions of Set1 and 2 primers are shown in Figure 4A. The PCR product of Set1 displayed no difference in the band intensity of mtDNA after knockdown of EndoG in HeLa cells (Figure 4B). Conversely, the mtDNA content of Set2 increased significantly by 28% after EndoG silencing (*p* = 0.0161), indicating EndoG-dependent mtDNA cleavage in control cells.

To examine the importance of EndoG's nuclease activity for the observed mtDNA removal, we analysed mtDNA amplification of Set2 after expression of EndoG-WT versus catalytically inactive mutant EndoG-H141A in HAP1 EndoG knockout cells. Whereas EndoG-WT expressing cells showed a decrease of Set2 mtDNA compared to control cells (average 29%, *p* = 0.0313), this was not the case with cells expressing the catalytically inactive mutant EndoG-H141A, (*p >* 0.9999) (Figure 4C).

We conclude that EndoG is involved in mitochondrial genome cleavage between positions 6134 and 15793 in a manner dependent on EndoG's nuclease activity. Given that we found EndoG's nuclease activity to be necessary for both the stimulation of replication initiation of mtDNA and for mtDNA cleavage, we next asked whether ROS induced oxidative damage may trigger these EndoG activities.

### ROS and oxidative lesions promote mtDNA cleavage by EndoG and replication initiation

**Figure 5:**
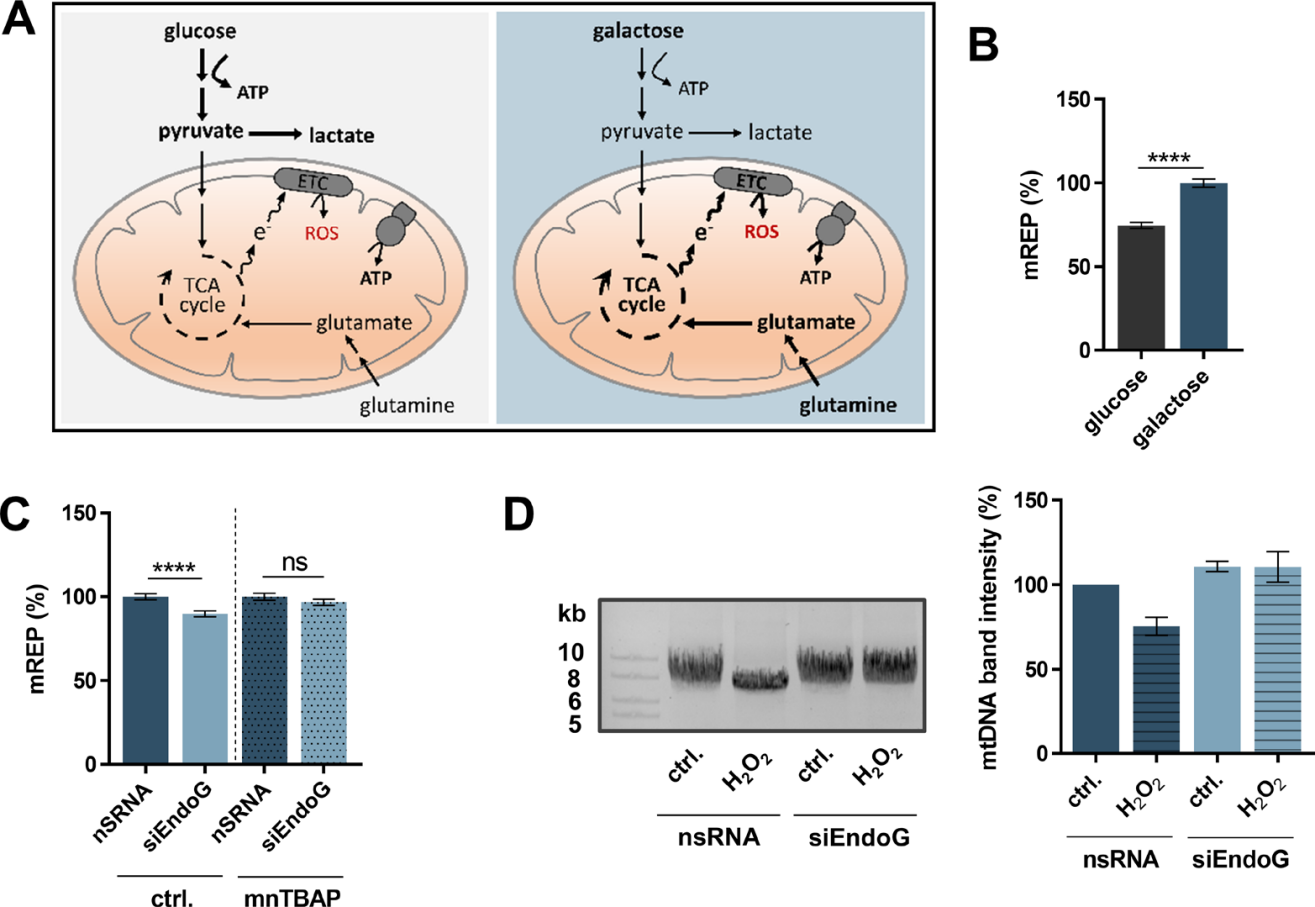
Impact of ROS-induced oxidative stress on EndoG's function in initiation of mtDNA O_H_ replication and mtDNA degradation (**A**) Graphical representation of cellular energy metabolism pathways of mammalian cells. Left panel: Cells grown in glucose medium produce ATP by means of OXPHOS as well as by metabolising glucose via glycolysis. Right panel: Galactose medium channels cells towards glutamine metabolism and enhances the activity of OXPHOS for ATP production instead of using glycolysis, with the consequence of an increased ROS formation (adopted from [47]). (**B**) Fluorescence-based quantification of mREP labelling (initiation of mitochondrial O_H_ replication) of HeLa cells grown in glucose or galactose medium for 72 h; *n* = 166–228 cells from 3 independent experiments. (**C**) Fluorescence-based quantification of mREP labelling of HeLa cells grown in galactose medium (72 h) and transfected with control nsRNA or siEndoG (48 h) and treated with either NaOH (ctrl.) or the ROS/RNS scavenger MnTBAP (100 μM) for 24 h; *n* = 214–225 from 3 independent experiments. Data are expressed as mean ± SEM. (ns, not significant; ^****^P < 0.0001; non-parametric Mann-Whitney test for unpaired samples). (**D**) Left panel: representative image of agarose gel electrophoresis of long-range PCR on total genomic DNA isolated from from HeLa cells grown in galactose medium (72 h) and treated with H_2_O (ctrl.) or 50 μM H_2_O_2_ for 5 h. Right panel: quantification of Set2 mtDNA band; *n* = 2 independent experiments; data are expressed as mean ± SD.

Mammalian cells are able to shift their energy metabolism, depending on the available type of sugar. Cells grown in glucose medium produce adenosine triphosphate (ATP) by OXPHOS and by metabolising glucose via glycolysis. When glucose is replaced with galactose, cells rely more on OXPHOS, which is a major source of ROS, than on glycolysis (Figure 5A) [47, 48]. We analysed the impact of the sugar metabolism and endogenous ROS levels on mtDNA replication. Replication initiation measured by mTRIP was significantly (1.3-fold) enhanced when cells were grown in galactose medium in comparison to cells grown in glucose medium (Figure 5B). This finding confirmed the notion that initiation of O_H_ replication is influenced by the mitochondrial metabolism. To gain a better understanding whether the increased initiation of O_H_ replication after EndoG knockdown is indeed ROS/reactive nitrogen species (RNS)-dependent and potentially linked to ROS/RNS induced damage, we treated HeLa cells with the ROS and RNS scavenger manganese(III) 5,10,15,20-tetrakis(4-benzoic acid)porphyrin (MnTBAP) [49, 50]. Highly toxic RNS are produced from harmless nitric oxide (NO) in the presence of ROS, therefore we did not limit this investigation to ROS. While EndoG knockdown revealed a significant decrease in replication initiation in galactose medium, this effect was eliminated after treatment with MnTBAP (Figure 5C). Therefore, we concluded that EndoG's function in replication initiation is at least in part dependent on ROS and/or RNS.

Since earlier work in yeast suggested a role of the EndoG ortholog in processing oxidative damage in mtDNA [51], we analysed the effect of enforced oxidative stress on mtDNA cleavage by EndoG. To this end we treated HeLa cells with H_2_O_2_ and measured the level of Set2 mtDNA with and without EndoG knockdown. H_2_O_2_ treatment (50 μM for 5 h) decreased the amount of Set2 mtDNA by 24%, but this decrease was completely abrogated when EndoG was silenced (Figure 5D). Thus, EndoG knockdown stabilises, at least in part, Set2 mtDNA, suggesting that EndoG cleaves mtDNA carrying oxidative lesions. To test the influence of BER (that repairs oxidative damage) in this process, we inhibited the key apurinic/apyrimidinic endonuclease 1 (APE1) enzyme via APE1inhibitorIII (APE1inhIII) and observed stabilisation of Set2 mtDNA (a 1.8-fold higher signal) by EndoG knockdown (Supplementary Figure 2A).

Given that oxidative stress exacerbated EndoG-mediated destabilisation of mtDNA, we wondered whether EndoG itself has an influence on cellular ROS or RNS levels. Therefore, we measured oxidation of 2’, 7’- dichlorofluorescin diacetate (DCF-DA) by ROS and Dihydrorhodamine 123 (*DHR123*) by RNS using fluorescence microscopy. EndoG knockdown induced the cellular ROS signal by 1.5-fold (Supplementary Figure 2B) as well as the RNS signal by 2.6-fold (Supplementary Figure 2C). As ROS is prevalently generated by the ETC in mitochondria, we evaluated the mitochondrial function by high-resolution respirometry using the OROBOROS Oxygraph-2k. Neither LEAK (the coupling efficiency) nor routine respiration nor complex I nor complex II activity showed significant alterations after EndoG knockdown (Supplementary Figure 2D). However, EndoG silencing induced a significant 1.3-fold increase in the maximal mitochondrial respiration (ETS, Supplementary Figure 2D), which could explain elevated ROS. Moreover, high ROS may also generate elevated RNS levels, via oxidation of NO after EndoG knockdown.

Taken together, our data are consistent with EndoG cleaving mtDNA carrying oxidative lesions and in turn modulating the nitroso-redox balance (RNS/ROS levels) in a negative feedback loop.

### Down-regulation of EndoG affects the expression of proteins involved in mtDNA replication

**Figure 6:**
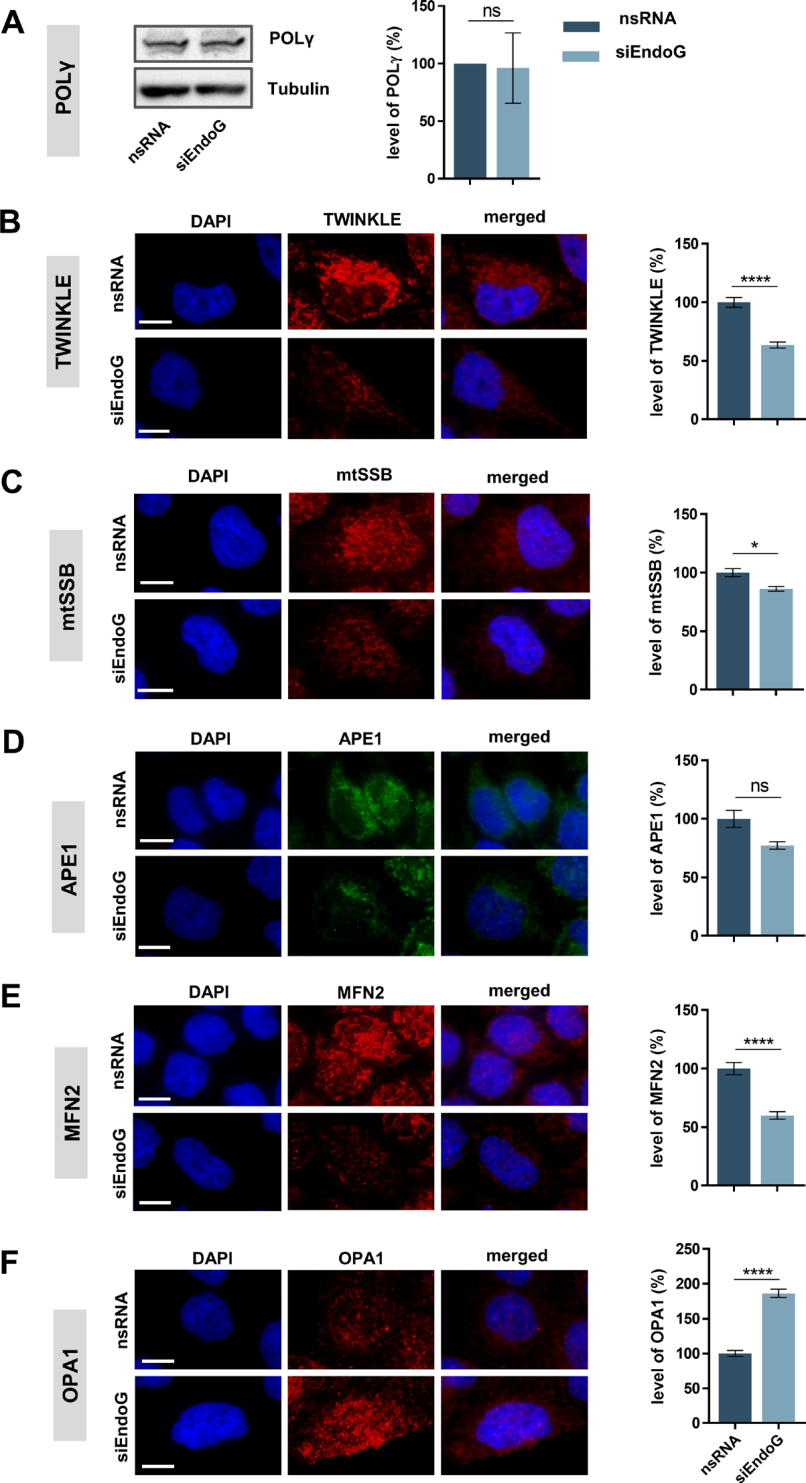
Impact of EndoG on expression of factors involved in mitochondrial genome replication, BER or fusion HeLa cells were grown in galactose medium (72 h) and transfected with control nsRNA or siEndoG and cultivated for 48 h. (**A**) Left panel: representative image of Western blot analysis of POLγ expression; right panel: quantification of Western blot analysis. *n* = 4 independent experiments. (B–F) Representative immunofluorescence microscopic images (left panel) and fluorescence-based quantification (right panel) of protein levels of (**B**) TWINKLE *n* = 120, (**C**) mtSSB *n* = 110–127, (**D**) APE1 *n* = 157–171, (**E**) MFN2 *n* = 157–171 or (**F**) OPA1 *n* = 103–141 cells from 2 independent experiments each. Data are expressed as mean ± SEM (ns, not significant; ^*^P < 0.05; ^****^P < 0.0001; non-parametric Mann-Whitney test for unpaired samples); Scale bars = 10 μm.

Our experiments demonstrated an involvement of EndoG in the removal of oxidatively damaged mtDNA. We speculated that this activity might lead to a compensatory induction of initiation of mtDNA O_H_ replication and, consequently, also to mitochondrial transcription. Consequently, we assessed whether EndoG affects factors known to be either part of the mitochondrial replication machinery or involved in BER. Western blot analysis revealed that EndoG knockdown did not alter the level of POLγ in HeLa cells (Figure 6A). Conversely, quantitative immunofluorescence microscopy indicated a significant decline of the helicase TWINKLE after EndoG knockdown by 37% (Figure 6B, *p <* 0.0001). mtSSB, which is also involved in both replication and BER [10, 52], was significantly reduced by 14% (Figure 6C, *p* = 0.0223). Moreover, EndoG knockdown did not significantly affect APE1 levels (Figure 6D, *p* = 0.9535). Thus, the mitochondrial replication machinery appears affected by EndoG silencing, whereas this may not be the case for the BER machinery.

Our observation of the influence of EndoG on the mitochondrial morphology (Supplementary Figure 1C) prompted us to also examine proteins involved in the fusion of mitochondrial membranes. First, we showed significant 40% reduction (*p <* 0.0001) of outer membrane fusion protein Mitofusin-2 (MFN2) after EndoG knockdown (Figure 6E), which is consistent with a previous study in H9c2 cardiomyocytes [53]. Conversely, the signal of the inner membrane fusion protein Optic atrophy-1 (OPA1) increased by 1.9-fold (Figure 6F, *p <* 0.0001). These results suggest improper balance of the fusion proteins upon EndoG knockdown.

## DISCUSSION

In this study, we investigated whether EndoG is involved in mitochondrial genome replication and maintenance in analogy with recently discovered nuclear functions of EndoG during replication stress [20, 54]. Our results show that EndoG promotes cleavage of mtDNA in consequence of oxidative and nitrosative stress, in turn inducing compensatory mtDNA replication.

Our initial finding of reduced replication initiation after EndoG knockdown was obtained by single-cell based mTRIP and confirmed by qPCR-based data. In this way, we substantiated an earlier proposal of a potential involvement of EndoG in the regulation of mtDNA replication [12]. In the literature mtDNA replication is reported to prematurely terminate at a high frequency resulting in the so-called 7S DNA molecule [7, 37]. 7S DNA forms a D-loop structure through hybridisation to the parental strand. The mechanism underlying D-loop formation may involve the TAS sequence at its 3´ boundary and a yet unknown TAS binding protein, which remains to be clarified [55, 56]. However, our qPCR data indicate that EndoG is not the sought-after candidate, as it did not differentially alter 7S DNA levels compared with longer versions of newly synthesised mtDNA. In this way a specific influence of EndoG on mtDNA replication via interfering with D-loop formation could likely be excluded.

We noticed that EndoG's role in the regulation of replication is dependent on its DNA binding and in particular its nuclease activity, since expression of corresponding deficiency mutant proteins did not stimulate mtDNA replication initiation. Moreover, our data indicate that EndoG mediates cleavage of mtDNA between positions 6134 and 15793 (Set2), possibly in the NCR (see below). This cleavage reaction was again dependent on EndoG's nuclease activity, suggesting cleavage of mtDNA by EndoG itself. Remarkably, EndoG-mediated cleavage of mtDNA was particularly pronounced in the presence of ROS or unrepaired base damage. Upregulation of mtDNA replication has previously been considered to represent a compensatory mechanism to cope with oxidatively damaged mtDNA [57, 58]. Interestingly, oxidative stress has been found to specifically affect the NCR region in HeLa cell mitochondria [59]. This region is enriched with guanine, which is a preferred target of oxidation, resulting in 8-oxo-G. We propose that EndoG´s role in upregulating mtDNA replication is an indirect outcome of its involvement in mtDNA degradation.

Our results are in agreement with work by Shokolenko *et al.* [45] revealing a decrease of mtDNA content after targeted accumulation of BER intermediates like apurinic/apyrimidinic (AP) sites in mtDNA. The authors suggested that while moderate oxidative mtDNA damage can be repaired by BER in mitochondria, mtDNA with more pronounced damage or excess repair intermediates will be degraded [45]. Here we demonstrate that EndoG is involved in this process of degradation, since in the absence of EndoG the cleavage reaction is diminished, which is particularly noticeable upon ROS exposure or APE1 inhibition. The fact that only a portion of mtDNA molecules are cleaved and degraded, as oxidative damage is primarily repaired by BER, can explain why, under unperturbed conditions, we observe only small changes in mtDNA levels as a function of EndoG. We therefore propose that EndoG is responsible for the cleavage reaction in severely oxidatively damaged mtDNA molecules or upon accumulation of the damage and BER intermediates. Conversely, none of the known mitochondrial nucleases, including EndoG, seem to be involved in massive mtDNA degradation, when DNA double strand breaks (DSBs) are introduced through expression of mitochondrially targeted restriction endonucleases [60].

Interestingly in 2014, Robertson *et al.* [61] demonstrated that EndoG preferentially cleaves double-stranded DNA with 5-hydroxymethylcytosine (5hmC) modifications of non C-phosphate-G (CpG) sites and thereby induces DSBs. 5hmC is produced by oxidative demethylation of 5-methylcytosine (5mC) catalysed by the three ten-eleven-translocation (Tet) enzymes (Tet1, 2 and 3) [62]. These modified cytosines are considered to some extent an epigenetic mark, involved in the regulation of gene expression of nuclear genes in the CpG context [63–67]. 5hmC was also found to colocalise with the DNA damage markers γH2AX and p53-binding protein 1 (53BP1) in cells undergoing replication stress [68]. Recently, evidence was provided for the existence of 5mC as well as 5hmC also in mtDNA [69], but the role of 5hmC, in both the mitochondria and the nucleus, is not yet fully understood [65, 70, 71].

In this context it is of interest that we recently discovered that during replication stress EndoG cleaves genomic DNA in the nucleus in a concerted action with activation-induced cytidine deaminase (AID) and the BER machinery [54]. Both AID and BER are known to be involved in the active demethylation process [71, 72]. Moreover, Zhang *et al*. [73] found that Tet2 converts 5mC to 5hmC during oxidative stress such as after H_2_O_2_ treatment, thereby triggering DNA demethylation. Given that oxidative stress activates Tet proteins [74] and knowing that EndoG cleaves 5hmC [61] in combination with our data indicating that EndoG-mediated cleavage of mtDNA is enhanced by oxidative stress, it is conceivable that Tet-dependent 5mC to 5hmC conversion triggers cleavage by EndoG. Thus, 5hmC may label mtDNA damage sites for EndoG-mediated cleavage, which is particularly relevant when oxidative damage as well as 5hmC cannot be efficiently corrected by BER and therefore need to be eliminated by degradation. We observed EndoG-dependent cleavage also when blocking the BER enzyme APE1, so that it is unlikely that the nucleases acting further downstream in BER, namely DNA replication helicase/nuclease 2 (DNA2), Flap endonuclease 1 (FEN1), Exonuclease G (ExoG) and MGME1 play a major role in this process. Ultimately the linearised damaged mtDNA molecules are most likely degraded, as mitochondria lack efficient DSB repair mechanisms [60]. In the nucleus EndoG-mediated cleavage activates DSB repair mechanisms instead of DNA degradation [20, 61, 75].

As outlined above our work reveals that mtDNA cleavage by EndoG is connected to increased replication initiation. Our experiments also provide evidence that ROS production stimulates replication initiation. Here, we used HeLa cervix cancer cells, which are known to shift to aerobic glycolysis for ATP production rather than OXPHOS (known as Warburg effect) [76–78]. By using galactose medium we could force cells to increase their OXPHOS activity [79] and thereby also ROS production, since it is known that the ETC is a major endogenous source of ROS [48, 80]. With galactose medium we saw enhanced replication initiation compared to glucose medium. Moreover, EndoG's impact on replication initiation was abolished in the presence of the ROS and RNS scavenger MnTBAP. Consistent with our hypothesis of compensatory mtDNA replication after 5hmC-mediated EndoG cleavage of oxidatively damaged mtDNA, work by Kowluru and Shan [81] revealed that MnTBAP antagonises hydroxymethylation as well as Tet2 binding to DNA. Compensatory replication may serve to balance the loss of mtDNA and concomitantly promote passive replication-dependent demethylation of mtDNA [82]. According to our protein expression data, two components of the mtDNA replication machinery, the nuclear-coded TWINKLE and mtSSB, are good candidates to mediate replication stimulation by EndoG.

While we observed downregulation of newly started heavy strand synthesis at O_H_ in the absence of EndoG, we did not observe significant alterations in 12S mtDNA levels. This apparent lack of correlation can be explained by the fact that 12S mtDNA is replicated late. It is possible that in the presence of EndoG, the number of initial, abortive molecules is larger than in the absence of the nuclease. This process could be linked to EndoG-dependent regulation of mtDNA content. Additionally, 12S mtDNA is part of Set2 targeted by EndoG-dependent degradation. In the absence of EndoG, reduced degradation may neutralise the effect of reduced synthesis resulting in apparently unaltered 12S mtDNA levels. Along this line, loss of mtDNA by EndoG might not be detectable in Set1 as it is synthesised early during compensatory mtDNA replication. A possible explanation for the fact that Set2 is preferentially removed by EndoG is that Set2 covers the mitochondrial NCR and the D-loop, i.e. hot spots of oxidative damage accumulation within mtDNA [83]. In this way especially Set2 might be a target for EndoG's cleavage of oxidatively damaged DNA. Noteworthy, 5hmC is enriched particularly in NCR mtDNA adjacent to 7S DNA [84], including the CSBII region, which is known to be preferentially attacked by EndoG [23].

While we carved out that EndoG acts downstream of oxidative stress, we also noticed that EndoG knockdown induces maximal mitochondrial respiration and ROS/RNS production. In the light of EndoG´s role in the removal of oxidatively damaged mtDNA the latter observation is in line with published work indicating that unrepaired, persistent single-strand breaks (SSBs) and mutations in mtDNA lead to an increase in ROS formation [85, 86]. In contrast to earlier findings, here under conditions of transient EndoG knockdown, we did not detect OXPHOS impairment or changes in respiration complex components, for which prolonged EndoG depletion might be necessary [44]. However, mtDNA depletion, compensatory replication and elevated mitochondrial respiration after EndoG knockdown were accompanied by morphological changes of mitochondria. We noticed fragmentation of elongated, contiguous mitochondrial assemblies into smaller, round and disconnected units. This observation can on the one hand be explained by the fact that upon activation of OXPHOS, mitochondria change from the so-called “orthodox” to the “condensed” morphology, which is also accompanied with cristae remodelling [87, 88]. In the absence of EndoG, increased fission could further serve to isolate most severely damaged mitochondria and direct them to degradation by selective mitophagy [89, 90]. At first sight paradoxically, we observed opposite effects of EndoG knockdown on MFN2 and OPA1 levels, even though both proteins are involved in mitochondrial fusion. However, MFN2 controls the fusion of the outer mitochondrial membrane, whereas OPA1 is needed for inner mitochondrial membrane fusion and independently plays a role in cristae remodelling [91]. Thereby OPA1 is able to modulate the stability of respiratory chain supercomplexes (RCS) and mitochondrial respiratory efficiency [92]. The imbalance between the two fusion factors may account for the altered morphology of mitochondria upon EndoG knockdown and elevated OPA1 additionally to mitochondrial respiration.

## CONCLUSIONS

**Figure 7:**
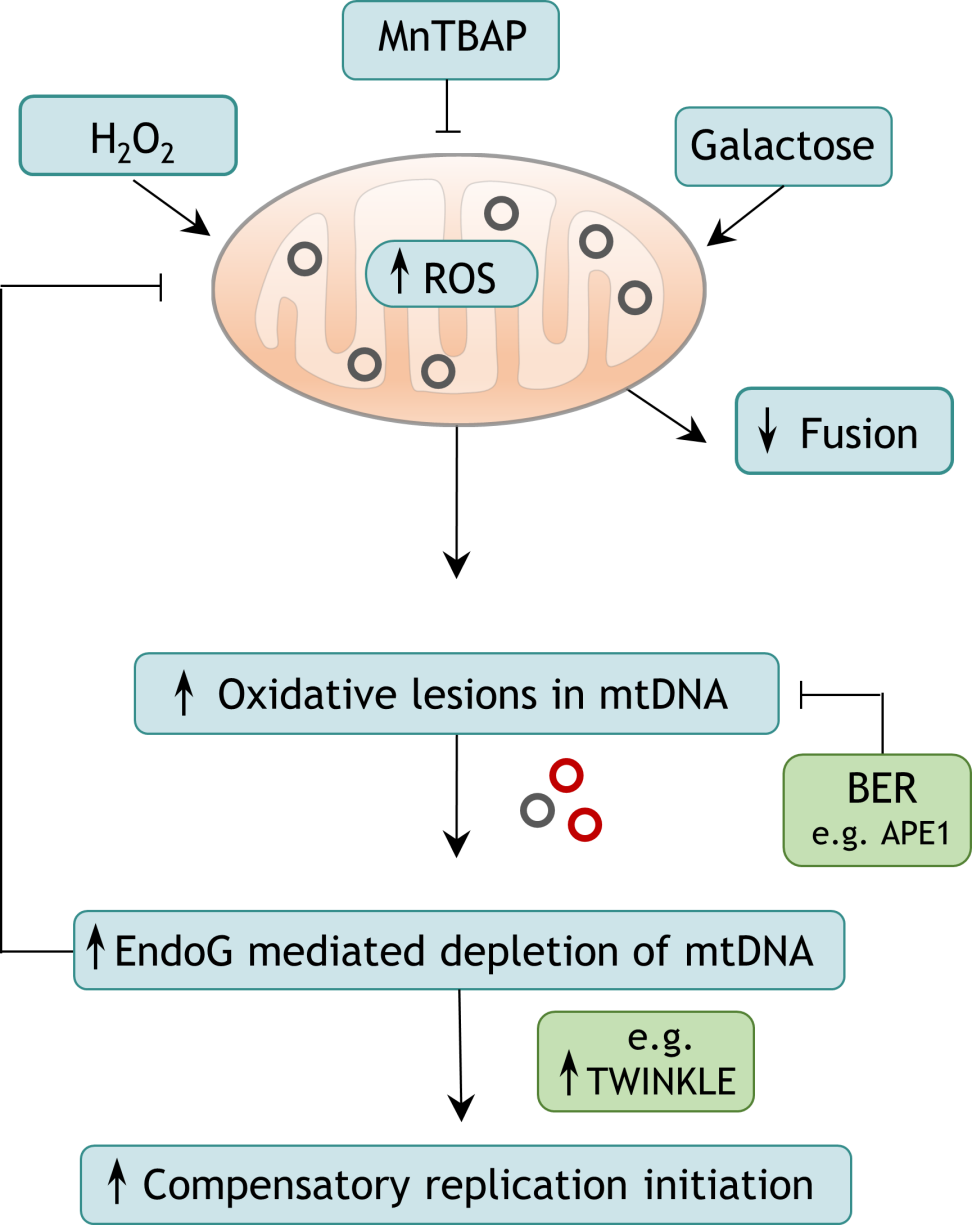
Proposed model of EndoG-mediated induction of mtDNA O_H_ replication initiation Elevated ROS levels, which mainly originate from the mitochondrial ETC and are supplied e.g. by galactose metabolism, cause oxidative damage to mtDNA. In case this damage is not repaired by BER, EndoG might perform a cleavage reaction. The resulting depletion of mtDNA possibly leads to compensatorily increased O_H_ replication initiation and downstream mitochondrial activities such as transcription.

In summary, EndoG is a key regulator of the mtDNA damage response and in this way a critical factor for mitochondrial genome maintenance. Our data provide evidence that EndoG cleaves mtDNA when oxidative lesions cannot be efficiently repaired. The resulting depletion of mtDNA results in compensatory mtDNA replication via expression of proteins involved in mtDNA replication, such as TWINKLE and mtSSB (Figure 7). We hypothesise that this cleavage reaction may be initiated by demethylation processes at non-CpG sites, providing a potential biological role for the yet unknown function of 5hmC accumulation in mtDNA. Since, changes in 5hmC mtDNA methylation are associated with a wide range of pathological conditions such as cancer, neurodegeneration and aging [65, 93], our observations broaden the current understanding of the development of these diseases and highlight EndoG as a potential target for future therapies.

## MATERIALS AND METHODS

### Cell culture

Human HeLa cells (provided by Heinrich-Pette-Institute, Hamburg, Germany) were propagated in DMEM medium (high glucose, 4mM L-glutamine, Gibco), supplemented with 10% foetal bovine serum (FBS) (Biochrom) and 2 mM L-glutamine (Gibco). Seventy-two hours before experiments HeLa cells were grown either in glucose or galactose medium as indicated. Galactose medium was composed of DMEM (without glucose, glutamine and phenol red), supplemented with 10% FBS, 50 mM galactose and 4 mM L-glutamine. HAP1 cells (Horizon Genomics, Vienna, Austria) were cultured in IMDM medium (Gibco), supplemented with 10% FBS. HAP1 is a near-haploid human cell line with fibroblast-like morphology, which was derived from the male chronic myelogenous leukemia (CML) cell line KBM-7 through cellular reprogramming [94]. All cells were grown at 37°C and 5% CO_2_.

### Plasmids and small interfering RNA (siRNA)

Plasmids for expression of EndoG-WT and for EndoG-H141A were purchased from OriGene (pCMV6-Entry Vector, RC205089). The EndoG-Δ-binding (EndoG-R133A-H141A-R184A-R188A) plasmid, which is based on the pLV-tetO-Oct4 plasmid (Addgene, 19766), was provided by J. Christof M. Gebhardt, Institute of Biophysics, Ulm University, Germany. Oct4 was replaced by the EndoG-WT or the mutated EndoG-Δ-binding sequence (3050–4852 nucleotides including a HALO-Tag^®^). pcDNA3.1 (Invitrogen) served as negative control. FlexiTube Gene-Solution GS2021 (Qiagen), which consists of a pool of four distinct siRNA, was used to transiently silence EndoG. Non-silencing RNA (nsRNA) (Qiagen, 1022076) served as negative control.

### Transfection and treatments

Different plasmids were transiently transfected into HAP1 cells by lipofection, using FuGENE^®^ HD Transfection Reagent (Promega). For siRNA-mediated EndoG knockdown in HeLa cells, HiPerFect^®^ Transfection Reagent (Qiagen) was utilised. Medium was changed 24 h after transfection and in case of ROS/RNS scavenger exposure cells were directly treated with 100 μM MnTBAP (Merck Millipore) or with the solvent NaOH (Fluka) for 24 h. For induction of oxidative stress 43 h post-transfection cells were exposed to 50 μM H_2_O_2_ (Fischar) *versus* H_2_O (Braun) for 5 h.

### Western blot

For preparation of HeLa and HAP1 lysates cell pellets were incubated in lysis buffer (50 mM Tris, pH 7.4; 150 mM NaCl; 2 mM EGTA; 2 mM EDTA; 25 mM NaF; 25 mM β-glycerophosphate; 0.1 mM NaV; 0.2% Triton X-100, 0.3% Nonidet P40), supplemented with protease inhibitor cocktail tablets (Roche). Protein concentrations were determined by means of the Pierce™ BCA Protein Assay Kit (Thermo Scientific). Afterwards 60 μg of protein were electrophoretically separated on 8–12% SDS-PAGE gels and blotted onto Hybond™-P 0.45 PVDF (GE Healthcare) membranes. Immunodetection was performed using mouse-anti-human DNA POLγ (Santa Cruz, sc-390634 [95]) and EndoG (Santa Cruz, sc-365359 [20]) primary antibodies. Mouse-anti-human α-tubulin (Abcam, ab7291 [96]) and Glyceraldehyde-3-phosphate-dehydrogenase (GAPDH, Abcam, ab9484 [97]) served as loading controls. Horseradish peroxidase (HRP)-conjugated goat-anti-mouse (Rockland) was utilised as secondary antibody. The peroxidase activity was visualised by Clarity™ Western ECL Substrate (Bio-Rad) and images were recorded with ChemiDoc™ MP (Bio-Rad). Quantification of band intensities was performed using Image Lab 4.1 (Bio-Rad). Each value of band intensity was corrected with the value of the corresponding loading control (α-tubulin or GAPDH).

### Immunofluorescence staining

HeLa cells were cultured on glass slides (12 mm, VWR), fixed with 3.7% Formaldehyde (AppliChem Panreac ITW companies), permeabilised with 0.5% Triton X-100 (Sigma-Aldrich), washed shortly with 0.05% Tween-20 (Merck) and blocked with 5% goat serum (Sigma-Aldrich) to avoid unspecific binding. For immunostaining slides were incubated for 1 h with the following primary antibodies (all from Santa Cruz): rabbit-anti-human mtSSB (sc-67101 [98]), TWINKLE (sc-134915 [99]) and APE1 (sc-334 [54]) as well as mouse-anti-human MFN2 (sc-100560 [100]) and OPA1 (sc-393296 [101]). Alexa Fluor 555 anti-mouse, Alexa Fluor 555 anti-rabbit and Alexa Fluor 488 anti-rabbit (all from Invitrogen) were used as secondary antibodies. HeLa cells were mounted with Vectashield^®^ containing 4′,6-Diamidin-2-phenylindol (DAPI, Vector laboratories) to stain the nuclei and imaged using a Keyence BZ-9000 microscope (Keyence Germany). The fluorescence intensity was analysed using the ImageJ 1.46/1.51 software (National Institutes of Health).

### Total genomic DNA extraction

Total genomic DNA was either extracted using the QIAamp^®^ DNA Mini Kit (Qiagen) or by classical isopropanol precipitation as follows: the cell pellet was incubated with 200 μl lysis buffer (0.2 mg/ml Proteinase K, 0.2% SDS, 5 mM EDTA in DPBS) for 3 h at 50°C. Afterwards 20 μl of 3M sodium acetate pH 5.2 and 300 μl of isopropanol (VWR Chemicals) were added to the lysates followed by incubation for 20min at −20°C and centrifugation (13000xg) for 30min at 4°C. Pellets were washed twice in 70% Ethanol (Honeywell) and resuspended in H_2_O (Braun). The total genomic DNA was quantified using NanoDrop 2000 Spectrometer (Thermo Scientific).

### Long-range PCR

In order to amplify the complete mitochondrial genome in two overlapping segments (Set1 and Set2), a special long-range PCR was performed [46]. 20–70ng of total genomic DNA were applied to the PCR reaction with LA Taq^®^ DNA Polymerase (TaKaRa). Primer sequences are described in Supplementary Table 1. 27–38 cycles were run at 94°C 1min, 94°C 30sec, 56°C 45sec, 68°C 11min, 72°C 10min for denaturing, annealing and elongation. PCR products were loaded on 0.5–0.8% agarose gels and imaged by ChemiDoc™ MP (Bio-Rad). Band intensity was quantified using the Image Lab™ (Bio-Rad) software or ImageJ 1.46 (National Institutes of Health).

### Probe labelling, denaturation and mTRIP imaging protocol

mTRIP was performed as previously described [34–36]. Briefly, mREP (marker of mitochondrial initiation of replication) and mTRANS (markers of mitochondrial transcripts) probes were amplified by PCR using 20ng of total genomic DNA. Primer sequences are represented in Supplementary Table 1. Cells were plated on slides, hybridised with mREP or mTRANS probes, washed with saline-sodium citrate (SSC), subsequently stained with Hoechst for 1 h or DAPI for 5min and washed with Dulbecco's phosphate-buffered saline (DPBS). Slides were mounted with 50% glycerol (Roth) in DPBS and imaged using either a LSM710 or a Zeiss Axiovert 200M confocal microscope. The fluorescence intensity was analysed with the ImageJ 1.34/1.46/1.51 software (National Institutes of Health).

### MitoTracker staining

Mitochondria were stained with 250–1000 nM MitoTracker^®^ Deep Red FM (MitoTracker DR, Life Technologies) at 37°C for 45 min −1 h and washed with medium and DPBS. Subsequently, cells were fixed with 2% Paraformaldehyde (PFA, Electron Microscopy Sciences), washed with DPBS and nuclei stained with 1 μg/ml DAPI (Sigma) for 5 min. After additional washing steps, slides were mounted with 50% glycerol (Roth)/DPBS. Images were taken using a LSM710 confocal microscope.

### Reverse transcription quantitative polymerase chain reaction (RT-qPCR) and qPCR

For RT-qPCR total mRNA was isolated from HeLa cells using the RNeasy^®^ Mini Kit, including DNase treatment, following reverse-transcription with QuantiTect^®^ Reverse Transcription Kit (all from Qiagen).

Total genomic DNA was employed for qPCR. The primers used (from Thermo Fisher and Biomers) are listed in Supplementary Table 1. Both RT-qPCR and qPCR were performed using KAPA SYBR^®^ FAST qPCR Kit (peqlab/VWR) on a Viia7 RUO thermocycler (Applied Biosystems Life Technologies). Data was analysed by Viia7 RUO software version 1.2.1. mRNA expression of target genes was calculated with the 2^−ΔΔCt^ method, whereby TATA box binding protein (TBP) transcript levels served as an internal control. For 7S and mtDNA analysis by qPCR the 2^ΔCt^ method was used and 18S rRNA served as internal control.

### Statistical analysis

Graphical presentation of data was performed by means of GraphPad Prism 7.03 (GraphPad Software Inc.) and statistical significance was analysed by using non-parametric Mann-Whitney test for unpaired samples or Wilcoxon matched-pairs signed rank test for related samples. ^*^P < 0.05; ^**^P < 0.01; ^****^P < 0.0001.

## SUPPLEMENTARY MATERIALS FIGURES AND TABLES



## Abbreviations

53BP1: p53-binding protein 1
AP: apurinic/apyrimidinic
APE1: apurinic/apyrimidinic endonuclease 1
APE1inhIII: APE1inhibitorIII
ATP: adenosine triphosphate
BER: base excision repair
C: complex
CML: chronic myelogenous leukemia
CSB: conserved sequence block
DCF: 2’, 7’- Dichlorofluorescein
DCF-DA: 2’, 7’- dichlorofluorescin diacetate
DHR123: Dihydrorhodamine 123
D-loop: displacement-loop
DNA2: DNA replication helicase/nuclease 2
DPBS: Dulbecco's phosphate-buffered saline
DTNB: 5, 5´dithiobis-2-nitrobenzoic acid
EndoG: Endonuclease G
ETC: electron transport chain
ERRα: Estrogen-related receptor alpha
ETS: maximal mitochondrial respiration
ExoG: Exonuclease G
FCCP: carbonyl cyanide-4-(trifluoromethoxy) phenylhydrazone
FEN1: Flap endonuclease 1
FISH: fluorescence *in situ* hybridization
Fwd: forward primer
H: heavy strand
GAPDH: Glyceraldehyde-3-phosphate-dehydrogenase
5hmC: 5-hydroxymethylcytosine
HSP: heavy strand promoter
HRP: Horseradish peroxidase
L: light strand
LSP: light strand promoter
MFN2: Mitofusin-2
DSBs: double strand breaks
MGME1: mitochondrial genome maintenance exonuclease-1
MitoTracker DR: MitoTracker Deep Red
*MLL*: Mixed-lineage leukemia
mtDNA: mitochondrial DNA
mTRIP: mitochondrial Transcription and Replication Imaging Protocol
MnTBAP: manganese(III) 5,10,15,20-tetrakis(4-benzoic acid)porphyrin
mtSSB: mitochondrial single-stranded DNA binding protein
NCR: non-coding region
NO: nitric oxide
ns: not significant
O_H_: origin of heavy strand replication
O_L_: origin of light strand replication
OPA1: Optic atrophy-1
OXPHOS: oxidative phosphorylation
PFA: Paraformaldehyde
PGC1α: Peroxisome proliferator-activated receptor gamma coactivator 1α
POLγ: polymerase γ
qPCR: quantitative polymerase chain reaction
RCS: respiratory chain supercomplexes
RNS: reactive nitrogen species
rev: reward primer
RITOLS: Ribonucleotide Incorporated ThroughOut the Lagging Strand
RNASEH1: ribonuclease H1
ROS: reactive oxygen species
RT-qPCR: Reverse transcription quantitative polymerase chain reaction
shRNA: small hairpin RNA
siRNA: small interfering RNA
SSB: single-strand break
SSC: saline-sodium citrate
TAS: termination associated sequences
TBP: TATA box binding protein
Tet: ten-eleven-translocation
TFAM: mitochondrial transcription factor A
TNB: 2-nitro-5-benzoic acid
WT: wild-type
