# Correction: Endonuclease G promotes mitochondrial genome cleavage and replication

**DOI:** 10.18632/oncotarget.25645

**Published:** 2018-06-12

**Authors:** Rahel Stefanie Wiehe, Boris Gole, Laurent Chatre, Paul Walther, Enrico Calzia, Anja Palmer, J. Christof M. Gebhardt, Miria Ricchetti, Lisa Wiesmüller

**Affiliations:** ^1^ Department of Obstetrics and Gynecology, Ulm University, Ulm, 89075, Germany; ^2^ Present address: Centre for Human Molecular Genetics and Pharmacogenomics, Medical Faculty, University of Maribor, Maribor, SI-2000, Slovenia; ^3^ Department of Developmental and Stem Cell Biology, Institute Pasteur, Stem Cells and Development, 75724 Cedex 15, Paris, France; ^4^ Team Stability of Nuclear and Mitochondrial DNA, Unit of Stem Cells and Development, CNRS UMR 3738, 75724 Cedex 15, Paris, France; ^5^ Central Facility for Electron Microscopy, Ulm University, Ulm, 89081, Germany; ^6^ Institute of Anesthesiological Pathophysiology and Process Engineering, Ulm University Hospital, Ulm, 89081, Germany; ^7^ Institute of Biophysics, Ulm University, Ulm, 89081, Germany

**This article has been corrected:** The correct author information and affiliations are given below:

Rahel Stefanie Wiehe^1^, Boris Gole^1,2^, Laurent Chatre^3,4^, Paul Walther^5^, Enrico Calzia^6^, Anja Palmer^7^, J. Christof M. Gebhardt^7^, Miria Ricchetti^3,4^ and Lisa Wiesmüller^1^

^1^ Department of Obstetrics and Gynecology, Ulm University, Ulm, 89075, Germany

^2^ Present address: Centre for Human Molecular Genetics and Pharmacogenomics, Medical Faculty, University of Maribor, Maribor, SI-2000, Slovenia

^3^ Department of Developmental and Stem Cell Biology, Institute Pasteur, Stem Cells and Development, 75724 Cedex 15, Paris, France

^4^ Team Stability of Nuclear and Mitochondrial DNA, Unit of Stem Cells and Development, CNRS UMR 3738, 75724 Cedex 15, Paris, France

^5^ Central Facility for Electron Microscopy, Ulm University, Ulm, 89081, Germany

^6^ Institute of Anesthesiological Pathophysiology and Process Engineering, Ulm University Hospital, Ulm, 89081, Germany

^7^ Institute of Biophysics, Ulm University, Ulm, 89081, Germany

Original article: Oncotarget. 2018; 9:18309-18326. https://doi.org/10.18632/oncotarget.24822

