# Supplementary material for: Endonuclease G promotes mitochondrial genome cleavage and replication
Source: Oncotarget. 2018 Apr 6;9(26):18309–26. doi: 10.18632/oncotarget.24822 (PMC5915074; doi:10.18632/oncotarget.24822)
Supplement: Supplementary file 1 [file oncotarget-09-18309-s001.pdf]

Endonuclease G promotes mitochondrial genome cleavage and replication

SUPPLEMENTARY MATERIALS

MATERIALS AND METHODS

Transfection and treatments

Transfection was performed as described in Material and Methods. For inhibition of Apurinic-apyrimidinic endonuclease 1 (APE1) 43 h post-transfection cells were exposed to 15 µM APE1-inhibitorIII (Merck/ Millipore) for 5 h. Controls were treated with DMSO (Merck Millipore).

MitoTracker staining

Mitochondria were stained with 250 nM MitoTracker® Deep Red FM (MitoTracker DR, Life Technologies) at 37°C for 45 min and washed with medium and DPBS, followed by the steps described in Material and Methods. Images were taken using a LSM710 confocal microscope and the fluorescence intensity analysed with the ImageJ 5.51 (Fiji) software (National Institutes of Health).

Immunofluorescence staining

Immunofluorescence staining was performed as described in Material and Methods. Slides were incubated for 1 h with mouse-anti-human EndoG primary antibody (Santa Cruz, sc-365359 [1]). Alexa Fluor 555 anti-mouse served as secondary antibody. Afterwards HeLa cells were mounted with Vectashield® containing DAPI (Vector laboratories) to stain the nuclei. Images were taken using a Keyence BZ-9000 microscope (Keyence Germany).

Mitochondrial quantification

To determine the mitochondrial content citric acid synthase activity was measured as previously described [2]. Briefly, 10 µg total protein was applied to reaction buffer (100 mM Tris HCl pH 8.1, 100 µM 5, 5′ dithiobis-2-nitrobenzoic acid (DTNB), 300 µM acetyl-CoA, 0.1% Triton X-100). Subsequent 500 µM oxaloacetate (Sigma Aldrich) was added as substrate for citric acid synthase and the conversion of DNTB to 2-nitro-5-benzoic acid (TNB) measured at a wavelength of 405nm.

DCF and DHR123 assay

To measure the nitroso-redox balance HeLa cells were treated either with 10 µM 2′, 7›-Dichlorofluorescin diacetate (DCF-DA, Sigma-Aldrich) or 10 µM DiHydroRhodamin 123 (DHR123, Sigma-Aldrich) at 37°C for 1 h. DCF-DA is oxidised to highly fluorescent 2′, 7′-Dichlorfluorescein (DCF) by ROS and DHR123 to Rhodamine123 mainly by RNS. Subsequently, cells were fixed with 2% PFA (Electron Microscopy Sciences), washed with DPBS and nuclei stained with Hoechst for 1 h or DAPI for 5 min. After an additional washing step, slides were mounted with 50% glycerol in DPBS and imaged using either a Keyence BZ-9000 microscope (Keyence Germany) or a Zeiss Axiovert 200M confocal microscope. Further the fluorescence intensity was analysed with the ImageJ 1.34/1.46 software (National Institutes of Health).

Western blot

Western blot was utilised as described in Material and Methods. 30–60 µg of protein were separated on 12% SDS-PAGE gels and blotted onto Hybond™-P 0.45 PVDF (GE Healthcare) membranes. Immunodetection was performed using a mouse-anti-human total OXPHOS antibody cocktail (Abcam, ab110411 [3]), recognizing the nuclear encoded NDUFB8 subunit of complex I, the SDHB subunit of complex II, the UQCRC2 subunit of complex III and the ATP5A subunit of complex V. Mouse-anti-human Glycerinaldehyd-3-phosphat-Dehydrogenase (GAPDH) (Abcam, ab9484) served as loading control. Horseradish peroxidase (HRP)-conjugated goat-anti-mouse (Rockland) was utilised as secondary antibody. The peroxidase activity was visualised by ClarityTM Western ECL Substrate (Bio-Rad) and images were recorded with ChemiDocTM MP (Bio-Rad). Quantification of band intensities was performed using the ImageJ 1.46 software (National Institutes of Health). Each value of band intensity was corrected with the value of the corresponding GAPDH loading control.

Electron microscopy

HeLa sells were seeded on carbon-coated sapphire discs. Fourty-eight hours after transfection with siRNA samples were high-pressure frozen using a Compact 01 HPF (Engineering Office M. Wohlwend GmbH) and freeze substituted according to Walther et al. [4]. Ultrathin sections were examined using a Jeol 1400 transmission electron microscope.

High-resolution respirometry

Mitochondrial respiration was monitored by high-resolution respirometry using OROBOROS Oxygraph-2k (OROBOROS Instruments Corp.). HeLa cells were grown in galactose medium for 72 h and transfected with siRNA against EndoG. After 48 h of EndoG knockdown, $0.5^{*}10^{6}$ HeLa cells/ml were added to the Oxygraph chamber in respiration medium containing 0.5 mM EGTA, 3 mM MgCl2.6H2O, 60 mM Lactobionic acid, 20 mM Taurine, 10 mM KH2PO4, 20 mM HEPES, 110 mM Sucrose, and 1 g/l bovine serum albumin and supplemented with 10 mM Pyruvate. Routine respiration was measured in the steady state level after closing the chambers, when oxygen consumption displayed a plateau. Afterwards oligomycin (400 µM) was added to inhibit ATP-synthase and obtain the LEAK respiration as an indicator of the coupling efficiency. To ascertain the maximal capacity of the electron transfer system (ETS) in the uncoupled state, the uncoupling agent carbonyl cyanide-4-(trifluoromethoxy) phenylhydrazone (FCCP) was gradually titrated into the mixture (6 × 125 nM). After permeabilization of the cell membrane with digitonin (2.4 µM), the activity of selected components of the ETC was determined by means of different substrates and inhibitors for the individual OXPHOS complexes. Complex I activity was determined by addition of 2 mM malate and 10 mM glutamate. Addition of cytochrome C (10 µM) assured the integrity of the outer mitochondrial membrane, as it changed the respiration by only 1–19%. Complex I + II activity was measured after addition of the substrate succinate (10 mM) and complex II after addition of complex I inhibitor Rotenone (0.25 µM). Finally, antimycin (5 µM) was used for inhibition of complex III. The measurements were analysed using DatLab version 5.1.0.20 (Oroboros Instruments Corp.).

REFERENCES

1. Cho HD, Lee JH, Moon KD, Park KH, Lee MK, Seo KI. Auriculasin-induced ROS causes prostate cancer cell death via induction of apoptosis. Food Chem Toxicol. 2018; 111:660–669.
2. Tews D, Fischer-Posovszky P, Fromme T, Klingenspor M, Fischer J, Rüther U, Marienfeld R, Barth TF, Möller P, Debatin KM, Wabitsch M. FTO deficiency induces UCP-1 expression and mitochondrial uncoupling in adipocytes. Endocrinology. 2013; 154:3141–51.
3. Tucker EJ, Hershman SG, Köhrer C, Belcher-Timme CA, Patel J, Goldberger OA, Christodoulou J, Silberstein JM, McKenzie M, Ryan MT, Compton AG, Jaffe JD, Carr SA, et al. Mutations in MTFMT underlie a human disorder of formylation causing impaired mitochondrial translation. Cell Metab. 2011; 14:428–34.
4. Walther P, Schmid E, Höhn K. High-pressure freezing for scanning transmission electron tomography analysis of cellular organelles. Methods Mol Biol. 2013; 931:525–35.
5. Chatre L, Ricchetti M. Prevalent coordination of mitochondrial DNA transcription and initiation of replication with the cell cycle. Nucleic Acids Res. 2013; 41:3068–3078.
6. Chatre L, Ricchetti M. mTRIP: an imaging tool to investigate mitochondrial DNA dynamics in physiology and disease at the single-cell resolution. Methods Mol Biol. 2015; 1264:133–47.
7. Rocheteau P, Chatre L, Briand D, Mebarki M, Jouvion G, Bardon J, Crochemore C, Serrani P, Lecci PP, Latil M, Matot B, Carlier PG, Latronico N, et al. Sepsis induces long-term metabolic and mitochondrial muscle stem cell dysfunction amenable by mesenchymal stem cell therapy. Nat Commun. 2015; 6:10145.
8. Suissa S, Wang Z, Poole J, Wittkopp S, Feder J, Shutt TE, Wallace DC, Shadel GS, Mishmar D. Ancient mtDNA genetic variants modulate mtDNA transcription and replication. PLoS Genet. 2009; 5:e1000474.
9. Antes A, Tappin I, Chung S, Lim R, Lu B, Parrott AM, Hill HZ, Suzuki CK, Lee CG. Differential regulation of full-length genome and a single-stranded 7S DNA along the cell cycle in human mitochondria. Nucleic Acids Res. 2010; 38:6466–6476.

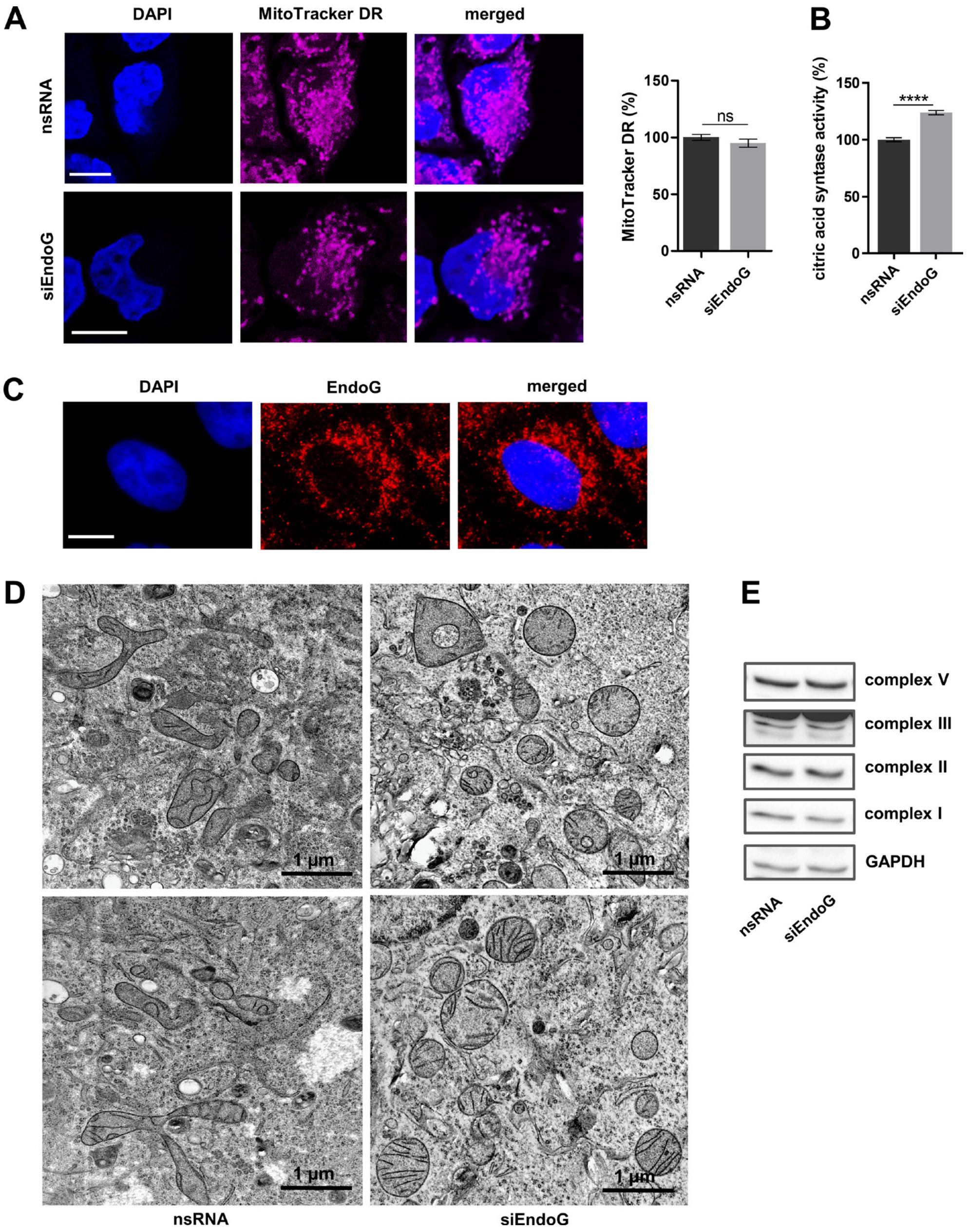

Supplementary Figure 1: Impact of EndoG on mitochondrial mass, morphology and expression of mitochondrial respiration complexes. HeLa cells were grown in glucose medium and transfected with control nsRNA or siEndoG and cultivated for 48 h. (**A**) Representative confocal microscopic images (left panel) and fluorescence-based quantification (right panel) of HeLa cells stained with DAPI and MitoTracker DR (250 nM, 45 min); n = 172–217 cells from 2 independent experiments; scale bars = 10 µM. (**B**) Quantification of mitochondrial content via citric acid synthase assay; n = 9 from 3 independent experiments. Data are expressed as mean ± SEM (ns, not significant; $^{****}P < 0.0001$; non-parametric Mann-Whitney test for unpaired samples). (**C**) Representative immunofluorescence microscopic images of EndoG signals in HeLa cells cultured for 72 h and stained with DAPI, scale bar = 10 µM. (**D**) Electron microscopic views of mitochondria. (**E**) Representative image of Western blot analysis. An OXPHOS antibody cocktail was used for immunoblotting, recognizing the nuclear encoded NDUFB8 subunit of complex I, the SDHB subunit of complex II, the UQCRC2 subunit of complex III and the ATP5A subunit of complex V; n = 3 independent experiments.

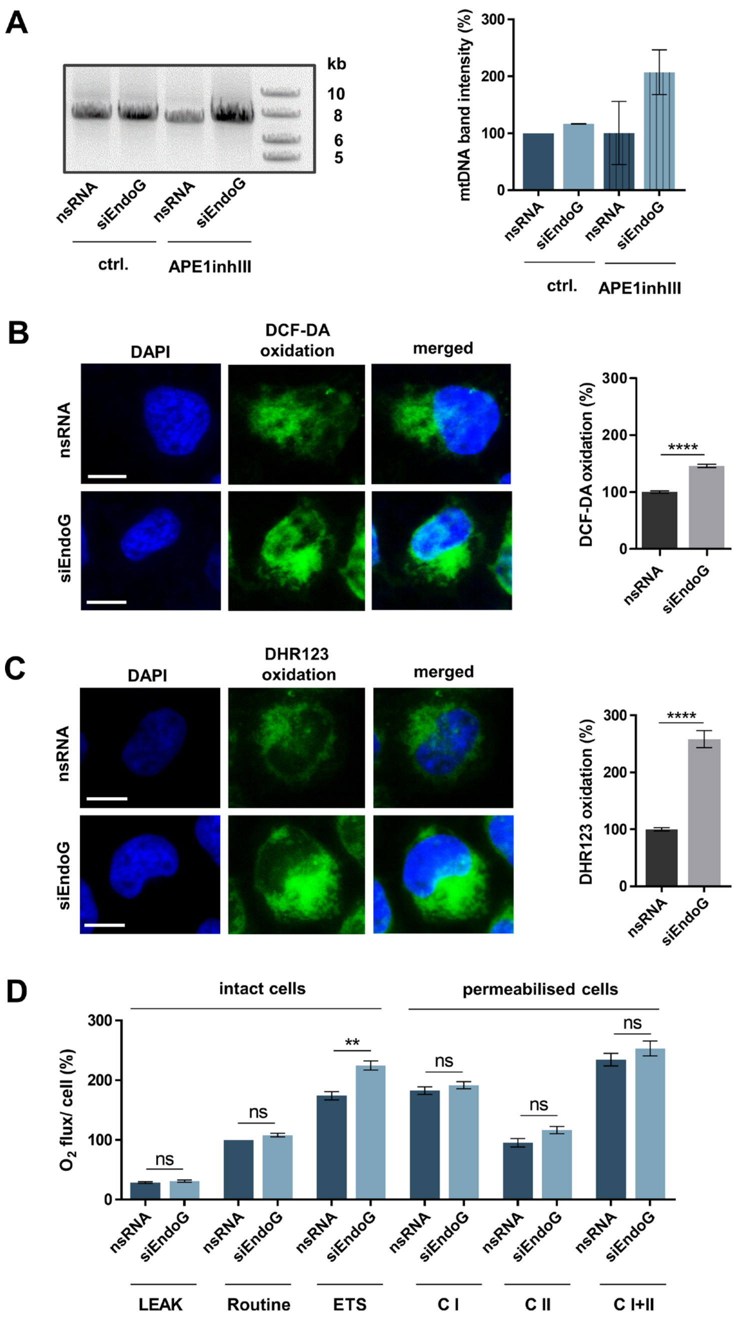

Supplementary Figure 2: Impact of BER inhibition for EndoG′s function in mtDNA degradation and the role of EndoG in regulating downstream mitochondrial activities. HeLa cells were transfected with control nsRNA or siEndoG and cultivated for 48 h. (**A**) Left panel: representative image of agarose gel electrophoresis of long-range PCR on total genomic DNA isolated from HeLa cells grown in galactose medium (72 h) and treated with DMSO (ctrl.) or 15 µM APE1inhIII for 5 h. Right panel: quantification of Set2 mtDNA band; $n = 2$ independent experiments, showing an increase after siEndoG knockdown (ctrl.); data are expressed as mean ± SD. For assessment of the nitroso-redox balance HeLa cells, grown in glucose medium, were either treated with 10 µM DCF-DA or DHR123 for 1 h. Representative microscopic images (left panel) and fluorescence-based quantification (right panel) of (**B)** oxidative stress (DCF-DA is oxidised to highly fluorescent DCF by ROS) and (**C**) nitrosative stress (DHR123 is oxidised to highly fluorescent Rhodamine123 mainly by RNS). n = 341–359 cells from 3 independent experiments; Data are expressed as mean ± SEM. (ns, not significant; $^{**}P < 0.01$, $^{****}P < 0.0001$; non-parametric Mann-Whitney test for unpaired samples); scale bars = 10 µM. (**D**) Comparison of respiration in HeLa cells, grown in galactose medium (72 h), characterised by mitochondrial oxygen flux per cell. Activity of selected components of the ETC was determined by treatment with different substrates and inhibitors for the individual OXPHOS complexes as detailed in Material and Methods ($n = 8$ independent experiments). Data are expressed as mean ± SEM. (ns, not significant; $^{**}P < 0.01$, $^{****}P < 0.0001$; (A–C) non-parametric Mann-Whitney test for unpaired samples; (D) Wilcoxon matched-pairs signed rank test for related samples). C - complex.

Supplementary Table 1: Primer sequences for amplification of mTRIP probes, long-range PCR, RT-qPCR and qPCR

Name	Size (bp)	Position in the mitochondrial genome	Primer sequence	Reference
**mTRIP**				
mREP	99	446–544	fwd 5′-ACATTATTTTCCCCTCCC-3′<br>rev 5′-GGGGTATGGGGTTAGCAG-3′	[5, 6]
mTRANS probe 1	962	1909–2870	fwd 5′-ACCAGACGAGCTACCTAAGAACAG-3′<br>rev 5′-CTGGTGAAGTCTTAGCATGT-3′	[5, 6]
mTRANS probe 6	1128	7406–8533	fwd 5′-CTACCACACATTCGAAGAACC-3′<br>rev 5′-CGTTCATTTTGGTTCTCAGGG-3′	[5, 6]
mTRANS probe 11	1421	13431–14851	fwd 5′-CATACCTCTCACTTCAACCTC-3′<br>rev 5′-TGAGCCGAAGTTTCATCATGC-3′	[5, 6]
**Long-range PCR:**				
Set1	10033	6037–16069	fwd 5′-GCAACCTTCTAGGTAACGACC-3′<br>rev 5′-GAGTCAATACTTGGGTGGTAC-3′	[7]
Set2	6910	15793–6134	fwd 5′-CATTGGACAAGTAGCATCCGT-3′<br>rev 5′-GCCTCCGATTATGATGGGTAT-3′	[7]
**RT-qPCR**				
16S	101	2627–2727	fwd 5′-GTATGAATGGCTCCACGAGG-3′<br>rev 5′-GGTCTTCTCGTCTTGCTGTG-3′	[5]
COII	110	8099–8208	fwd 5′-ACAGATGCAATTCCCGGACG-3′<br>rev 5′-GGCATGAAACTGTGGTTTGC-3′	[5, 8]
ND6	114	14258–14371	fwd 5′-ATTGGTGCTGTGGGTGAAAG-3′<br>rev 5′-GGATCCTCCCGAATCAACCC-3′	[5]
TBP	80	-	fwd 5′-CTCACAGGTCAAAGGTTTAC-3′<br>rev 5′-GCTGAGGTTGCAGGAATTGA-3′	[5]
**qPCR**				
7S A-B1	80	16180–16259	A: fwd 5′-GTGGCTTTGGAGTTGCAGTT-3′<br>B1: rev 5′-AAAACCCCCTCCCCATGCTTA-3′	this work
7S A-B2	224	16036–16259	A: fwd 5′-GTGGCTTTGGAGTTGCAGTT-3′<br>B2: rev 5′-GAAGCAGATTTGGGTACCAC-3′	[5, 9]
12S	153	1130–1282	fwd 5′-GCTCGCCAGAACACTACGAG-3′<br>rev 5′-CAGGGTTTGCTGAAGATGGC-3′	[5]
18S	105	-	fwd 5′-GAGAAACGGCTACCACATCC-3′<br>rev 5′-GCCTCGAAAGAGTCCTGTAT-3′	[5, 8]

The probe name, the start and end point of PCR amplification in the mitochondrial genome, the size of PCR products, the sequence of primers as well as references are indicated. Homo sapiens mitochondrion, complete genome sequence NC_012920.1 (Gene Bank) was used as reference. Fwd – forward primer, rev – reward primer.
